# Bidirectional Relationship Between Sickle Cell Disease and Food Insecurity: Scoping Review

**DOI:** 10.1089/heq.2023.0147

**Published:** 2024-04-03

**Authors:** Faeben Wossenseged, Kristina Franklin, Talya Gordon, Ashley Buscetta, Gwenyth R. Wallen, Vence L. Bonham, Nicole Farmer

**Affiliations:** ^1^ Social and Behavioral Research Branch, National Institutes of Health, National Human Genome Research Institute, Bethesda, Maryland, USA.; ^2^ Translational Biobehavioral and Health Disparities Branch, National Institutes of Health, Clinical Center, Bethesda, Maryland, USA.

**Keywords:** sickle cell disease, food insecurity, chronic disease, nutrition security, diet, health disparities

## Abstract

**Introduction:**

In the United States, sickle cell disease (SCD)—the homozygous inheritance of a point mutation within the beta-globin chain of hemoglobin—affects between 80,000 and 100,000 people. Adequate nutrition can influence the pathophysiology of SCD, and individuals with SCD who are undernourished are more likely to have impaired immune function and disease exacerbation. Undernourishment is often caused by food insecurity (FI), which is defined as “a household-level economic and social condition of limited or uncertain access to adequate food” by the USDA. FI disproportionately affects African Americans, a population disproportionately affected by SCD in the United States.

**Objectives:**

We performed a scoping review to better understand the relationship between FI and SCD severity.

**Methods:**

A comprehensive search for peer-reviewed research articles and meeting abstracts was conducted in accordance with Preferred Reporting Items for Systematic Reviews and Meta-Analyses (PRISMA) guidelines. Selected studies were reviewed for descriptive analysis by three independent reviewers.

**Results:**

In total, 72 studies were identified, 62 were excluded for meeting inclusion criteria. The remaining 10 studies, 5 of which were meeting abstracts, were reviewed. Although limited evidence is available, the results of this scoping review suggest a bidirectional relationship between SCD and FI. Seven key themes were identified to help elucidate this relationship: 1) prevalence of FI among individuals with SCD, 2) child versus caregiver experiences of FI, 3) psychosocial factors, 4) food assistance benefits, 5) dietary intake, 6) external spending, 7) healthcare utilization.

**Conclusion:**

Findings from this scoping review suggest how SCD and FI work in tandem to exacerbate each other. Furthermore, the findings illustrate current gaps in the literature and opportunities for actions to address FI among individuals living with SCD.

## Background

In the United States, sickle cell disease (SCD) affects between 80,000 and 100,000 people, disproportionately impacting African Americans.^1^ Individuals who live with SCD suffer from complications such as debilitating pain, fatigue, infections, and end-organ damage, as well as psychosocial complications.^2,3^ While advances for SCD are being made, such as gene therapy and the approval of the medications voxelotor and crizanlizumab, there is emerging research on how nutrition can influence the pathophysiology and outcomes of SCD.^4,5^ Literature has described the nutritional implications of SCD, including micronutrient and macronutrient deficiencies, higher nutrient energy needs, and growth abnormalities.^6–8^ Individuals with SCD who are undernourished are more likely to have impaired immune function, poor ulcer healing, and disease exacerbation.^8^

Nutrition reflects the intersection and balance between dietary intake and physiologic need. However, nutrition also reflects social determinants of health (SDoH)—the social, cultural, and economic circumstances and environments to which an individual is exposed during their lifetime.^9^ These determinants can coalesce and impact nutrition through the development of food insecurity (FI).^10^ According to the USDA, FI is defined as “a household-level economic and social condition of limited or uncertain access to adequate food,” affecting ∼12% of U.S. households and 8% of children.^11,12^

The term was coined in the 1970s to measure food supply. Since then, “food insecurity” has been operationalized using various metrics and has evolved to measure access to food supply and necessary nutrients, food availability, food utilization, or temporal or situational factors.^13^ In addition, significant heterogeneity still exists in the measurement of FI due to variability of cross-cultural dietary habits, the price of commodities, and the distribution of food in a household.^14^ In the United States, The Bureau of the Census developed an instrument that can assess FI called the “US Household Food Security Survey Module.”^14,15^

African Americans are the predominant ancestral group among individuals living with SCD in the United States. African Americans with SCD may have health care outcomes impacted by structural racism.^16^ Defined as the system of discriminatory policies that assign opportunity and access on the basis of race, structural racism can impact those with SCD through housing insecurity, health care access resources, interpersonal discrimination in health care settings, and disparities in food environments.^16^ The unequal impact of FI within African American households may be reflective of structural racism.^17^ A disproportionate number of residential areas in African American communities are marked by inaccessibility to affordable and healthy foods. These communities are often referred to as “food deserts,” “food swamps,” or “food apartheid zones.”^18^ Within the United States, food accessibility in low-income census tracts is mapped using the Food Access Research Atlas.^19^

In addition to representing social inequities, FI may also contribute to and reflect health inequities.^20^ Seligman et al. have reported a bidirectional relationship that may occur between FI and health outcomes among individuals living with chronic disease, including resultant psychosocial stress and inadequate nutritional intake.^21^ Current understanding of adverse psychosocial factors in health outcomes for individuals with SCD suggests identifying FI among this population may be important.^6^

To identify the impact of FI among individuals with SCD, a scoping review was conducted. The objectives of this scoping review were to address the following questions: (1) What is the current evidence on FI in the literature among individuals living with SCD? (2) What gaps and limitations exist in the literature concerning FI among individuals living with SCD?

## Methods

The scoping review was conducted in accordance with Preferred Reporting Items for Systematic Reviews and Meta-Analyses (PRISMA) guidelines.^22^ The search strategy for this review was created in consultation with a research librarian and a comprehensive search for peer-reviewed articles was conducted using the following databases: PubMed, Embase, Cochrane, CINAHL, Scopus, and Web of Science. References within the articles were examined for additional articles (both forward and backward reference searching).

The database, references, and communications with the librarian were conducted and searched in November 2020. The search consisted of all articles published from 2010 to November 2020. This broad timeframe was selected due to the limited number of articles published that contained studies aimed at exploring FI in individuals with SCD. Using the same search terms and databases as in the first search (Appendix I), updated searches were conducted in July 2021 and January 2022 for all articles and abstracts published during December 2020–July 2021 and July 2021–January 2022, respectively.

All identified literature were screened for inclusion. The inclusion criteria were as follows: (1) written or translated in English; (2) measured food security/insecurity; and (3) included individuals recruited from an SCD patient or family population. There was no demographic (e.g., adults and pediatric population, race, and gender) or geographic specificity. Article eligibility was determined by reviewing the title and abstract from the research results. If the article's relevance was not apparent from the title and abstract, the article was retrieved and read. Details of screening results are included in Appendix S2 PRISMA 2020 checklist Health Equity.

A total of three database searches were conducted between December 2020 and January 2022. From all of the database searches, 72 articles were identified and 65 remained after duplications were excluded. Thirty of the remaining articles were not reviewed as they did not meet the inclusion criteria. From the initial search, five articles were then reviewed independently as a pilot by three researches (F.W., K.F., T.G.) for descriptive analysis. The publication dates of the selected articles ranged from 2018 to 2020 and included populations from the United States and Brazil. From all databases searches, a reference search of each article was conducted, but no further articles for inclusion were identified.

**FIG. 1.**
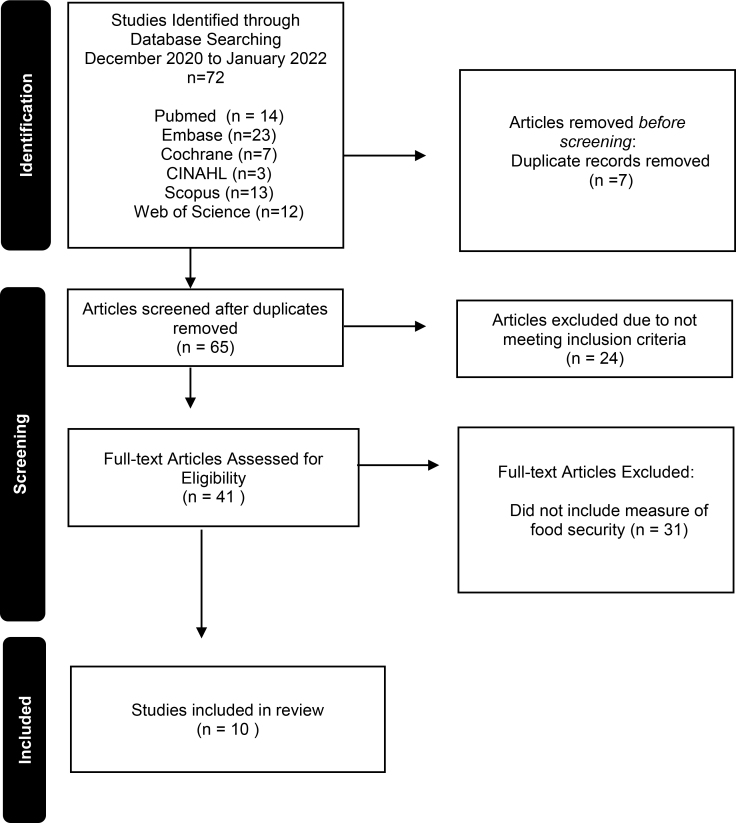
Illustration of the process of searching and selecting relevant articles included in the scoping review.

For the second search, seven articles were identified and five remained after duplications were excluded. Four articles were not reviewed due to failure to meet inclusion criteria. Authors conducted direct search on Embase and extracted three articles. In total, four new abstracts were found and reviewed in the second literature search process (Fig. 1). For the third search, two articles were identified through PubMed, one of them being the published article of an abstract that was previously reviewed. Both articles were reviewed.

The results of the screenings were compared and discussed by creating a data extraction table (Table 1). The table was used to capture relevant information across the articles reviewed. Data was extracted by K.F., F.W., and T.G.. The information captured were the general study design/methods, study type, study population and sample size, and main findings/results. After reviewing the studies independently, the main characteristics and findings were abstracted and grouped according to common themes describing contributing factors influencing the relationship between FI and SCD. This process involved content analysis of data from the extraction table through grouping of findings based on commonalities across studies.

**Table 1.** Summary of Themes and Main Findings

Themes	Findings
Prevalence of FI among individuals with	62.2% (*n*=190) (Santos et al. 2019)
SCD	62% (*n*=27) (Agamah et al. 2019)
	40.6% (*n*=101) (Power-Hays et al. 2020)
	35% (*n*=107) (Gruntorad et al. 2018)
	21.3% (*n*=99) (Ghafuri et al. 2020)
	30% (*n*=100) (Fernandez et al. 2022)
	34% (*n*=100) (Supples et al. 2019)
	35% (*n*=99) (Darlington et al. 2021)
	33.5% (*n*=523) (Khan et al. 2020)
	8.4% (youth), 13.2% (caregivers) (*n*=100) (Green et al. 2022)
Child vs. caregiver experiences of food insecurity	45.8% of children with SCD reported food insecurity, whereas only 21.3% of these children's caregivers reported food insecurity (Ghafuri et al. 2020). 8.4% of children with SCD reported food insecurity compared to 13.2% of their caregivers (Green et al. 2022)
Psychosocial factors	Inverse relationship between social support and food insecurity (Santos et al. 2019) Direct association between QOL and severity of SCD among children (Gruntorad et al. 2018) Higher FI was associated with lower QOL scores. QOL scores were inversely associated with healthcare utilization (Darlington et al. 2021)
Food assistance benefits	80% of participants received at least one federal food assistance benefit (Fernandez, 2022) 52% of participants received SNAP benefits in the last month (Darlington et al. 2021) 53% of participants received SNAP benefits (Gruntorad et al. 2018) 60.5% of participants received government social benefits (Santos et al.2019)
Dietary intake	48% of adult participants screened negative for food insecurity, 80% of those who screened positive did not consume the daily recommended dietary allowance of vegetables and fruits (Agamah et al. 2019). Children experiencing food insecurity, with or without housing instability, had significantly higher intake of dairy and pizza compared to children living with no economic instability. Children experiencing both food insecurity and housing instability had lower intake of whole grains compared to children only experiencing housing instability alone (Fernandez et al. 2022). Food insecurity was associated with higher pizza intake among children (Fernandez et al.). No significant difference in body mass index (BMI) was found between food insecure and food secure pediatric participants (Ghafuri et al.).
External spending	Association found between mild food insecurity and severity of SCD could possibly be a result of increased health expenditures due to SCD, which can then affect a family s total budget, influencing their food security (Santos et al. 2019). 44% of food insecure participants were worried about having enough money for food and stated that the cost of healthy food was a barrier to eating healthy (Agamah et al. 2019).
Health care utilization	Pediatric participants with SCD and Household Material Hardship (HMH) had higher Emergency Department utilization than those without HMH (Power-Hays et al. 2020). Food insecure participants had significantly higher prevalence of acute chest syndrome, missed clinic visits, and active opioid prescriptions (Supples et al. 2019). Food insecure participants had higher rates of ED visits and hospitalization, though they were not statistically significant (Supples et al. 2019). No significant differences in morbidity incidence for acute severe vaso-occlusive pain and acute chest episodes requiring hospitalization (Ghafuri et al.). Children with SCD living in households without vehicles and >0.5 miles from a supermarket had higher rates of hospitalization and acute care utilization (Khan et al. 2020). Ability to predict SCD-related acute events among pediatric patients by the age of 6 significantly improved when poor food access was considered in the predictive model (Khan et al. 2020).

ACS, acute chest syndrome; ACU, acute care utilization; BMI, body mass index; ED, emergency department; HMH, household material hardship; QOL, quality of life; SNAP, Supplemental Nutrition Assistance Program.

## Results

The 10 studies included in the scoping review were classified into common themes describing contributing factors influencing the relationship between FI and SCD. The titles of these themes are as follows: Prevalence of FI among Individuals with SCD; External Spending; Psychosocial Factors; Nutritional Intake; and Child versus Caregiver Experiences of FI. Themes were organized according to the frequency of appearance in each study with the most common theme listed first. Table 1 presents the summary of each theme and their main findings.

### Study population

The study populations in this scoping review included female and male participants from the United States and Brazil. The study participant characteristics provided by each study are provided in Table 2.

**Table 2.** Study Populations of the Reviewed Studies

Author	Study population
Agamah (2019)	Adult SCD population at the UIHHSS in Chicago, Illinois 29.6% male, 70.3% female
Supples et al. (2019)	Pediatric SCD population at Brenner Children's Hospital in Winston-Salem, North Carolina <18 years old
Fernandez et al. (2022)	Pediatric SCD population and parents at Pediatric Sickle Cell Center of Columbia University Irving Medical Center in New York City, New York 55% male, 45% female 1–21 years old Mean age of 10.6±5.6 years 85% HbSS, 13% HbSC, 3% HbSB0
Santos et al. (2019)	Pediatric SCD population and their families at a referral hematology hospital in Rio de Janeiro, Brazil 5–9 years old
Khan et al. (2020)	Pediatric SCD population recruited from Sickle Cell Clinical Research and Intervention Program 59.3% male, 51.7% female Median age of 5.5 years (range 1–6)
Ghafuri et al. (2020)	Pediatric SCD population and their caregivers in Nashville, Tennessee 46.7% male, 53.3% female Median age of 10.4 69.3% HbSS or HbSβ0
Gruntorad et al. (2018)	Pediatric SCD population from The CSCDRG clinical registry 2–24 years old 79% Hgb SS, 16% Hgb SC, and 5% Hgb Sβ+/0
Syed et al. (2021) (same study population as Gruntorad et al.)	48% male, 52% female 5–24 years old 76% Hgb SS
Power-Hays et al. (2020)	Families of children with SCD 12 months of age and up to, but not including, 18 years, treated at BMC in Boston, Massachusetts 52.5% male, 47.5% female Mean age of 8.7 74.3% with HbSS genotype
Green et al. (2022)	50 caregiver-youth dyads enrolled in HABIT trial in New York City and Philadelphia 10–18 years old −41.7% female, 58.3% male (youth), 81.6% female, 18.4% (caregivers)

BMC, Boston Medical Center; CSCDRG, Chicago Sickle Cell Disease Research Group; HABIT, Hydroxyurea Adherence for Personal Best in Sickle Cell Treatment; SCD, sickle cell disease; SS; UIHHSS, University of Illinois Hospital and Health Science Systems.

Of note, Gruntorad et al. and Syed et al. used the same study population, with the former's included age range being 5–24 years, and the latter's being 2–24 years.^23,24^

### Study measurements

As shown in Table 3, measures of FI varied across the reviewed studies. Ghafuri et al. and Fernandez et al. used the 18-item U.S. Household Food Security Survey Module (household level).^25,26^ The questionnaire includes a series of statements that seek to assess whether a household could afford the food they need in the past 12 months.^27^ After completing the questionnaire, answer choices are coded, with the number of affirmative answer choices (i.e., yes, sometimes, often, almost every month, some months but not every month) counted. A key is provided to convert the raw score into level of FI, with two different keys: one for families with no children and one for families with one or more children.

**Table 3.** Reviewed Studies, Study Types, and Study Measurements

Authors	Abstract or full length article	Study types	FI measurements
Agamah (2019)	Abstract	Cross-sectional and exploratory study	FIES
Ghafuri et al. (2020)	Full length	Single-center cross-sectional study	18-item U.S. Household Food Security Survey Module 9-item Youth Food Security Survey Module
Gruntorad et al. (2018) Syed et al. (2021)	Abstract Abstract	Prospective cohort study (baseline results are presented)	USDA Food Security Short Form
Power-Hays et al. (2020)	Full Length	Single-center retrospective study	SDoH screener
Santos et al. (2019)	Full Length	Cross-sectional study	Brazilian FI Scale
Supples et al. (2019)	Abstract	Cross-sectional study	The Hunger Vital Sign
Khan et al. (2020)	Abstract	Cross-sectional study	U.S. Food Access Research Atlas
Fernandez et al. (2022)	Full Length	Cross-sectional study	18-item U.S. Household Food Security Survey Module
Green et al. (2022)	Full Length	Cross-sectional study	The Hunger Vital Sign

FI, food insecurity; FIES, Food Insecurity Experience Scale; SDoH, social determinants of health.

To capture responses from children 12 years of age or older, Ghafuri et al. used the adapted 9-item Youth Food Security Survey Module.^25^ This adapted module from the U.S. Household Food Security Survey Module asks questions about the food situation and the child's eating behavior in the home during the previous month.^27^

Power-Hays et al. used the SDoH screener developed by Boston Medical Center (BMC) network in Massachusetts to assess FI.^28^ The SDoH screener is a self-reported, paper questionnaire completed by the families of pediatric patients with SCD during their pediatric hematology clinic appointments. The BMC SDoH screeners usually integrate electronic health records, community support, and referral resources.^29^ A participant was considered having positive household material hardship (HMH) if they answered “Yes” to any question about paying for housing, food, transportation, and utilities or requested connection to resources on the SDoH screener.^29^ FI is determined by affirmative responses to “Within the past 12 months, the food you bought didn't last and you didn't have money to get more” and “Within the past 12 months, you worried whether your food would run out before you got money to buy more.”^29^

Agamah et al. measured FI using the Food Insecurity Experience Scale (FIES) from the Food and Agriculture Organization (FAO).^30^ FIES is experience-based measures of household or individual food security. The FIES Survey Module consists of eight questions regarding people's access to adequate food.^31^ Severity of FI is measured on an increasing scale, starting from “worrying about running out of food” on the mild end, “compromising on quality and variety,” “reducing quantities, skipping meals,” and finally “experiencing hunger” on the severe end.^31^

Gruntorad et al. and Syed et al. utilized the USDA Food Security Short Form to collect data on the food security status of patients.^23,24^ This survey is a six-item questionnaire created by the National Center for Health Statistics. It is a modified shortened version of an 18-item module, aimed to reduce response burden, and is able to identify FI and very low food security.^32^

Supples et al. and Green et al. utilized the Hunger Vital Sign, a two-question FI measurement adapted from the U.S. Household Food Security Survey Module.^33,34^ The two questions within the survey concern financial worries associated with buying food within the past year.^35^

Rather than directly surveying participants using an FI screening tool, Khan et al. measured food access using home addresses of participants mapped to census-tract environmental data using the U.S. Food Access Research Atlas, to determine if participants lived in areas deemed as food deserts.^19,36^

Santos et al., the only international study within this review, utilized the Brazilian FI Scale. This scale consists of 14 yes-no questions assessing household FI over the past 90 days. Households are categorized as food secure, mildly food insecure, moderately food insecure, and severely food insecure.^37,38^

### Themes

#### Prevalence of FI among individuals with SCD

FI prevalence among participants with SCD estimates ranged from 21.3% to 62.2% in all studies, as seen in Table 1. Using the FIES, Agamah et al. reported that 40% screened moderate, 22% screened as severe for FI, and the remaining 38% were food secure.^30^ Power-Hays et al. found that 40.6% of participants were food insecure.^28^ In addition, 68.3% were FI and possessed one or more HMH.^28^ Santos et al. noted that 62.2% of families of children with SCD interviewed were food insecure.^38^ Gruntorad et al. discovered 35% of the participants with SCD interviewed were food insecure and 53% utilized public food benefit programs.^23^

The highest rates of FI were observed among patients 5–7 (57%) and 13–17 (42%) years of age.^23^ Syed et al., whose study population consisted of a subsection of Gruntorad's, also found 35% reported FI, with 52% of participants utilizing public food benefit programs.^24^ The highest rates of FI were found among patients 8–12 (30%) and 13–17 (27%) years of age.^24^ Ghafuri et al. observed that within a cohort of 24 pediatric patients diagnosed with sickle cell disease, a notable 45.8% of respondents reported instances of household FI.^25^

Fernandez et al. discovered that 30% of families with children diagnosed with sickle cell disease experienced food insecurity, while 80% received at least one form of federal food assistance benefit.^26^ Similarly, Supples et al. revealed that 34% of families with children diagnosed with SCD experienced food insecurity, while Khan et al. reported that 33.5% of the children in their study with sickle cell disease resided in census tracts classified as “food deserts.”^33,36^ Green et al. observed that among their sample of 50 caregiver-youth dyads, 8.4% of pediatric patients and 13.2% of caregivers reported experiencing FI.^34^

### Child versus caregiver experience of FI

Ghafuri et al.'s study highlighted a discordance between caregiver- and child-reported FI, with 45.8% of children with SCD reporting FI, in comparison to 21.3% of these children's caregivers.^25^ In addition, evidence was found that FI in households of children with SCD (based on adult responses) was higher compared to the national U.S. average.^25^ Green et al. also reported a discordance between caregiver- and child-reported FI of 8.4% of youth versus 13.2% of caregivers. This difference was potentially attributed to social desirability.^34^

### Psychosocial relationship (quality of life±social support as a protective factor)

Santos et al. found an inverse relationship between social support and FI, specifically among multiple dimensions of social support, including informational, social interaction, and tangible dimensions.^38^ Gruntorad et al. assessed the quality of life (QOL) scores of SCD patients by age group, measuring the following dimensions: pain and hurt, pain management, pain impact, worry, treatment, and communication. Total average QOL scores for the cohort was 76 and highest QOL scores were measured among patients 2–4 years of age, indicating a better QOL, while lowest scores were measured among those 18 years of age or older.^23^ Syed et al.'s study found that lower QOL scores were associated with higher rates of FI. QOL scores were inversely associated with ER visits, hospital stays, and nights of hospitalization.^24^

### Food assistance benefits

Several studies documented federal food assistance benefits received by participants, with 80% receiving at least one from the study conducted by Ghafuri et al., 52% receiving Supplemental Nutrition Assistance Program benefits within the last year in Syed et al.'s study, and 53% in Gruntorad et al.'s study (no set amount of time specified).^23–25^ Santos et al. found that 60.5% of participants received government social benefits.^38^

### Dietary intake

Agamah et al. detected in their study that, although 48% of adult participants screened negative for FI, 80% of all participants did not consume the daily recommended dietary allowance of vegetables and fruits.^30^ In addition, Fernandez et al. found that pediatric and young adult patients who were experiencing FI and house instability had an increased pizza and dairy intake compared to food and housing secure patients. Also, food and housing insecure patients had lower whole grain intake compared to patients only experiencing housing instability. These findings significantly associate FI with poorer diet quality.^26^ Ghafuri et al.'s was the only study to measure the body mass index (BMI) of study participants. Although a direct correlation has been found between obesity and FI, no significant difference in BMI was found between food secure and food insecure participants.^25^

### External spending

External spending due to SCD can be defined as health expenditures toward SCD treatment and income loss due to SCD-related reasons, such as productivity loss due to pain crisis or hospital stays.^39^ Only one study in our review directly reported economic costs as a factor for FI. Agamah et al. noted that 44% of their participants were concerned about having enough money for healthy food.^30^ Although not directly measured, Santos et al. remarked that the association found between mild FI and severity of SCD could possibly occur due to increased health expenditures which can then affect a family's total budget.^38^

### Health care utilization

Power-Hays et al. explored the relationship between HMH and pediatric SCD patient reliance on the emergency department (ED). It was found that pediatric participants with SCD and HMH had higher ED utilization than those without HMH and were more likely to have visited the ED in the prior year. Importantly, the total emergency department reliance (EDr) was 12% and the EDr increased by an average of 7.7 points per additional HMH. However, while those with a housing hardship had an EDr that was 12.6 percentage points greater than those without and those with a utility hardship had an EDr 11.7 percentage points greater than those without, participants with FI did not have a difference in average EDr.^28^

Supples et al. identified a significantly higher prevalence of acute chest syndrome (ACS), missed clinic visits, and opioid prescriptions among food-insecure individuals, suggesting that their food insecurity status could potentially contribute to adverse health outcomes.^33^ Ghafuri et al. discerned no significant difference in acute severe vaso-occlusive pain and ACS leading to hospitalization between food-secure and food-insecure pediatric participants.^25^ Finally, Khan et al. demonstrated that the predictive ability for SCD-related acute events by the age of 6 significantly enhanced when poor food access was incorporated within the predictive model.^36^ In addition, children with SCD living in households without vehicles and living >0.5 miles from a supermarket had increased hospitalization and acute care utilization, while living >1 mile was associated with a high risk of hospitalizations.^36^

## Discussion

Although limited evidence in the literature is available, the results of this scoping review suggest a significant prevalence and impact of FI among individuals living with SCD. Our review noted several outcomes associated with FI, identified psychosocial factors that may mitigate or adversely affect health outcomes, and identified areas of further research such as measurement tools, ideal populations to screen, and impact on dietary intake, stressing the importance of nutritional security related to SCD.

**FIG. 2.**
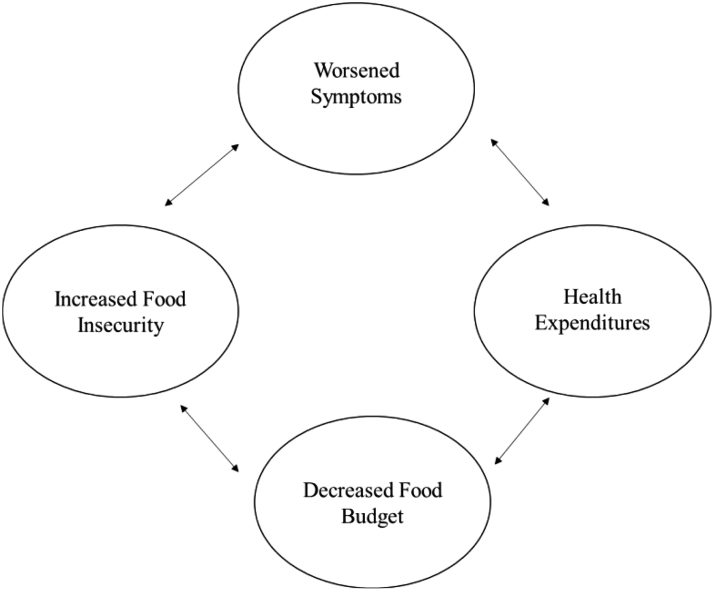
Bidirectional relationship between FI and SCD severity (worsened symptoms) with mechanistic factors included (decreased food budget and health expenditures). FI, food insecurity; SCD, sickle cell disease.

Our findings help to establish a bidirectional relationship between SCD severity and FI, as first conceptualized by Seligman et al.^21^ FI among adults can lead to less consumption of foods that contain essential nutrients.^40^ Given the role of key nutrients such as iron, zinc, copper, and folic acid in the pathophysiology of SCD, FI may thus greatly impact disease severity. Simultaneously, an increase in disease severity results in a need for more treatment, increasing health expenditures and resulting in decreased financial resources available for nutritious foods (Fig. 2).^21^ Potential factors that our review identified within the bidirectional relationship include dietary intake, QOL, and social support.

### Dietary intake

In conceptualizing the bidirectional relationship between FI and SCD, it is important to consider the direct connection between diet and health. As previously mentioned, diet plays an important role in the pathophysiology of SCD. However, Agamah et al. found that 80% of the adult participants with SCD did not consume the daily recommended dietary allowance of vegetables and fruits, regardless of FI status.^30^ Nutritional intake among individuals with SCD has been reported to be inadequate in general; deficiencies in micronutrients such as iron, zinc, copper, folic acid, pyridoxine, and vitamin E are prevalent in patients with SCD and are associated with increased disease severity of SCD.^7^ In addition, it has been found that food insecure adults eat less vegetables, fruit, and dairy products and have lower intake of vitamins A and B-6, calcium, magnesium, and zinc.^21^

### Quality of life

Although a majority of SCD health expenditures within the United States are covered by Medicaid, the financial burden also extends itself to general income and productivity loss.^39,41^ Individuals with SCD often experience chronic comorbidities and symptoms, such as pain, vaso-occlusive crises, depression, and fatigue, which can affect their ability to work.^28,33,36^ In addition, individuals with chronic diseases have been found to have lower QOL scores, with SCD patients experiencing even lower QOL scores in comparison, with greater reports of pain, lower productivity, and worse health in general.^42^ FI has also been found to impact QOL, with children from low-income households having lower health QOL scores.^43^ How QOL scores may be associated with FI status among SCD patients remains to be explored, particularly in how SCD contributes to limiting financial budget, time, and mobility when it comes to food access.

### Social support

Santos et al. identified an inverse relationship between FI and social support, with social support providing families of SCD pediatric patients with a means of improving their economic status or coping with the stress of FI.^38^ This theory aligns with the stress-buffering hypothesis, which states that social support reduces the harmful effects of negative stressors by allowing individuals to redefine the harm of their situation, increasing their perceived ability to cope, and providing more avenues for possible solutions.^44^ Overall, social support is an important asset in minimizing the disproportionate physical and psychosocial risks of FI and SCD.

### Limitations

There are several limitations that must be accounted for when interpreting this scoping review. First, most of the available literature is based within the United States, providing a limited understanding of the possible relationship between SCD and FI. In addition, no national registry for individuals with SCD exists in the United States or Brazil, limiting the accurate assessment of the prevalence of FI among the total SCD populations. Variable information regarding study participants were included across studies, limiting our ability to fully identify factors related to FI.

Across 10 studies in our review, 8 measurement tools were utilized, resulting in the collection of inconsistent information, and limiting our ability to fully identify factors related to FI. Furthermore, although a systematic search strategy and screening was used in our study, it is possible that some articles evaluating FI among people with SCD were not screened. Finally, most of the literature included within this scoping review are abstracts that have not yet been peer reviewed and may not present the full findings of the studies. Therefore, the level of evidence is weakened by their presence, and shows the need to move the literature to full peer-reviewed research articles.

### Call to action

FI is a complex and multifaceted issue, and without attempts to better measure and address it, the optimization of health-related QOL in patients and their families is compromised. Future efforts should be conducted to educate researchers, physicians, and caregivers of children with SCD of their children's dietary requirements. As recommended by Power-Hays and McGann, universal screening for SDoH should be instituted.^16^

We also propose assessing SDoH screening in unison with FI screening. Attention to FI within SCD clinical research is also needed. Inclusion of FI screening in larger cohorts of pediatric and adult SCD patients is necessary to assess the rates of FI more accurately in this population and to measure the impact on disease progression. Finally, FI within SCD should be contextualized by the nutritional factors needed for disease management, such as dietary intake quality, energy intake, and macronutrient content. In this way, consideration of tools that are structured through the lens of nutritional insecurity may be beneficial.

## Conclusion

FI among SCD individuals is an underresearched topic, with scant literature exploring the bidirectional relationship between FI and SCD. In this review, we aimed to explore the current state of the literature and highlight the need for future research addressing FI in the SCD population. The stress and financial strain associated with living with a chronic disease such as SCD may tighten food budgets, which consequently limits access to necessary nutritious food. This bidirectional loop may lead to SCD individuals with FI having more severe episodes of pain, which may exacerbate financial and psychosocial stressors, and further increase health inequities. As the importance of diet in relation to SCD continues to be explored, clinicians must consider the barriers to food access and the potential for FI that their patients may be experiencing.

## Supplementary Material

Supplemental data

Supplemental data

## Supplementary Material

Supplementary Appendix S1

Appendix S2 PRISMA 2020 Checklist Health Equity.docx

## Abbreviations Used

ACS: acute chest syndrome
ACU: acute care utilization
BMC: Boston Medical Center
BMI: body mass index
CSCDRG: Chicago Sickle Cell Disease Research Group
ED: emergency department
EDr: emergency department reliance
FAO: Food and Agriculture Organization
FI: food insecurity
FIES: Food Insecurity Experience Scale
HABIT: Hydroxyurea Adherence for Personal Best in Sickle Cell Treatment
HMH: household material hardship
PRISMA: Preferred Reporting Items for Systematic Reviews and Meta-Analyses
QOL: quality of life
SCD: sickle cell disease
SDoH: social determinants of health
SNAP: Supplemental Nutrition Assistance Program
UIHHSS: University of Illinois Hospital and Health Science Systems
