# Supplementary material for: Bidirectional Relationship Between Sickle Cell Disease and Food Insecurity: Scoping Review
Source: Health Equity. 2024 Apr 3;8(1):238–48. doi: 10.1089/heq.2023.0147 (PMC11002324; doi:10.1089/heq.2023.0147)
Supplement: Supplemental data [file Suppl_AppendixS1.docx]

Appendix 1

**Search Strategy Documentation**

**Database: PubMed
Provider: U.S. National Library of Medicine**

**Date(s) Searched: Dec 17, 2020
Date range searched: 2010 - 2020**

**#1)** "food security"[tiab] OR "food insecurity"[tiab] OR food insecurity[mh] OR food security[majr] OR "food access"[tiab] OR "food hardship"[tiab] OR "food availability"[tiab] OR "food assistance"[tiab] OR "food desert"[tiab] OR "food deserts"[tiab] OR food deserts[mh] OR "food supply"[tiab] OR "food supplies"[tiab] OR "food insecure"[tiab] OR "food secure"[tiab] OR "food environment"[tiab] OR "food banks"[tiab] OR "food bank"[tiab] OR "food pantries"[tiab] OR "food pantry"[tiab] OR food supply[majr] OR "supplemental assistance nutrition program"[ti] OR "nutrition access"[tiab] OR supermarket[tiab] OR supermarkets[tiab] OR "grocery stores"[tiab] OR "grocery store"[tiab] OR "corner stores"[tiab] OR "convenience stores"[tiab] OR "food store"[tiab] OR groceries[tiab] OR "retail food environment"[tiab] OR “food retail”[tiab] OR "food marts"[tiab] OR "food mart"[tiab] OR “food outlets"[tiab] OR "food outlet"[tiab] OR “food environment”[tiab] OR “food environments”[tiab] OR “food mirages”[tiab] OR “fast-food”[tiab] OR “fast food”[tiab] OR food assistance[mh] OR "food aid"[tiab] OR "food stamp*"[tiab]

AND

**#2)** "sickle cell"[tiab] OR anemia, sickle cell[mh] OR "hemoglobin s disease*"[tiab] OR "Hbs disease"[tiab] OR "sickle cell anemias"[tiab] OR "sickle cell anemia"[tiab] OR "Sickle Cell Anemias"[tiab] OR "Hemoglobin S"[tiab] OR "Hemoglobin S Diseases"[tiab] OR "Sickle Cell Disorders"[tiab] OR "Sickle Cell Disorder"[tiab] OR "HbS Disease"[tiab] OR "Sickle Cell Disease"[tiab] OR "Sickle Cell Diseases"[tiab]

**#3)** #1 AND #2

Filters: Humans, English

­­­­­­­­­­­­­­

**Database**: **Embase
Provider: Elsevier**

**Date(s) Searched: December 17, 2020
Date range searched: 2010 to 2020**

**#1 -** "food security":ti,ab OR "food insecurity":ti,ab OR "food access":ti,ab OR "food hardship":ti,ab OR "food availability":ti,ab OR "food assistance":ti,ab OR "food desert":ti,ab OR "food deserts":ti,ab OR "food supply":ti,ab OR "food supplies":ti,ab OR "food insecure":ti,ab OR "food secure":ti,ab OR "food environment":ti,ab OR "food banks":ti,ab OR "food bank":ti,ab OR "food pantries":ti,ab OR "food pantry":ti,ab OR food supply:ti,ab OR "nutrition access":ti,ab OR supermarket:ti,ab OR supermarkets:ti,ab OR "grocery stores":ti,ab OR "grocery store"[taib] OR "corner stores":ti,ab OR "convenience stores":ti,ab OR "food store":ti,ab OR groceries:ti,ab OR "retail food environment":ti,ab OR “food retail”:ti,ab OR "food marts":ti,ab OR "food mart":ti,ab OR “food outlets":ti,ab OR "food outlet":ti,ab OR “food environment”:ti,ab OR “food environments”:ti,ab OR “food mirages”:ti,ab OR “fast-food”:ti,ab OR “fast food”:ti,ab OR 'food insecurity'/exp OR 'food access'/exp OR 'food availability'/exp OR 'food security'/exp OR "food aid":ti,ab OR "food stamp*":ti,ab

**#2** - 'sickle cell':ti,ab OR 'sickle cell anemia'/exp OR "hemoglobin sc disease*":ti,ab OR "Hbs disease":ti,ab OR "Hemoglobin S Diseases":ti,ab

**#3** - #1 AND #2

**#4** - #4 AND [humans]/lim AND [english]/lim

**Database: CINAHL
Provider: EBSCOhost
Date(s) Searched: December 17, 2019
Date range searched: 2010 - 2020**

**#1** - (MH "Food Security") OR "food security" OR (MH "Food Assistance") OR "food security" OR "food insecurity" OR "food access" OR "food hardship" OR "food availability" OR (MH "food supply") OR "food assistance" OR "food desert" OR "food deserts" OR "food supply" OR "food supplies" OR "food insecure" OR "food secure" OR "food environment" OR "food banks" OR "food bank" OR "food pantries" OR "food pantry" OR "food supply" OR "supplemental assistance nutrition program" OR wic OR "nutrition access" OR supermarket OR supermarkets OR "grocery stores" OR "grocery store" OR "corner stores" OR "convenience stores" OR "food store" OR groceries OR "retail food environment" OR “food retail” OR "food marts" OR "food mart" OR “food outlets" OR "food outlet" OR “food environment” OR “food environments” OR “food mirages” OR “fast-food” OR “fast food”

**#2** - "sickle cell" OR (MH "anemia, sickle cell") OR "hemoglobin s disease*" OR "Hbs disease" OR "sickle cell anemias" OR "sickle cell anemia" OR "Hemoglobin S" OR "Hemoglobin S Diseases" OR "Sickle Cell Disorders" OR "Sickle Cell Disorder" OR "HbS Disease"

**#3** - #1 AND #2

Filters: English Language, Human

**Database: Cochrane Database of Systematic Reviews
Provider:** John Wiley & Sons

**Date(s) Searched: December 17, 2020
Date range searched: 2010 – 2020**

**#1 -** "food security" OR "food insecurity" OR [mh "food insecurity"] OR "food access" OR "food hardship" OR "food availability" OR "food assistance" OR "food desert" OR "food deserts" OR "food supply" OR "food supplies" OR "food insecure" OR "food secure" OR "food environment" OR "food banks" OR "food bank" OR "food pantries" OR "food pantry" OR [mh "food supply"] OR "supplemental assistance nutrition program"[ti] OR "nutrition access" OR supermarket OR supermarkets OR "grocery stores" OR "grocery store" OR "corner stores" OR "convenience stores" OR "food store" OR groceries OR "retail food environment" OR "food retail" OR "food marts" OR "food mart" OR "food outlets" OR "food outlet" OR "food environment" OR "food environments" OR "food mirages" OR "food mirage" OR "fast-food" OR "fast food" OR "food aid" OR "food stamp*"

**#2 -** "sickle cell" OR [mh "anemia, sickle cell"] OR "hemoglobin s disease*" OR "Hbs disease" "Hemoglobin S" OR "Hemoglobin S Diseases"

**#3 -** #1 AND #2

Limits: Trials, English, Humans

**Database: Scopus
Provider:** Elsevier

**Date(s) Searched: December 17, 2020
Date range searched: 2010 – 2020**

**#1 -** "food security" OR "food insecurity" OR "food access" OR "food hardship" OR "food availability" OR "food assistance" OR "food desert" OR "food deserts" OR "food supply" OR "food supplies" OR "food insecure" OR "food secure" OR "food environment" OR "food banks" OR "food bank" OR "food pantries" OR "food pantry" OR "supplemental assistance nutrition program" OR "nutrition access" OR supermarket OR supermarkets OR "grocery stores" OR "grocery store" OR "corner stores" OR "convenience stores" OR "food store" OR groceries OR "retail food environment" OR "food retail" OR "food marts" OR "food mart" OR "food outlets" OR "food outlet" OR "food environment" OR "food environments" OR "food mirages" OR "food mirage" OR "fast-food" OR "fast food" OR "food aid" OR "food stamp*"

**#2 -** "sickle cell" OR "hemoglobin s disease*" OR "Hbs disease" "Hemoglobin S" OR "Hemoglobin S Diseases"

**#3 -** #1 AND #2

Limits: English, Humans

**Database: Web of Science
Provider:** Clarivate Analytics

**Date(s) Searched: December 18, 2020
Date range searched: 2010 – 2020**

**#1** - TOPIC: ("food security" OR "food insecurity" OR "food access" OR "food hardship" OR "food availability" OR "food assistance" OR "food desert" OR "food deserts" OR "food supply" OR "food supplies" OR "food insecure" OR "food secure" OR "food environment" OR "food banks" OR "food bank" OR "food pantries" OR "food pantry" OR food supply OR "supplemental assistance nutrition program" OR "nutrition access" OR supermarket OR supermarkets OR "grocery stores" OR "grocery store" OR "corner stores" OR "convenience stores" OR "food store" OR groceries OR "retail food environment" OR “food retail” OR "food marts" OR "food mart" OR “food outlets" OR "food outlet" OR “food environment” OR “food environments” OR “food mirages” OR “fast-food” OR “fast food” OR "food aid" OR "food stamp*")

**#2** - TOPIC: ("sickle cell" OR "sickle cell anemia" OR "hemoglobin s disease*" OR "Hbs disease" OR "sickle cell anemias" OR "Hemoglobin S" OR "Hemoglobin S Diseases" OR "Sickle Cell Disorders" OR "Sickle Cell Disorder" OR "HbS Disease" OR "Sickle Cell Disease" OR "Sickle Cell Diseases")

("sickle cell" OR "sickle cell anemia" OR "hemoglobin s disease*" OR "Hbs disease" OR "sickle cell anemias" OR "Hemoglobin S" OR "Hemoglobin S Diseases" OR "Sickle Cell Disorders" OR "Sickle Cell Disorder" OR "HbS Disease" OR "Sickle Cell Disease" OR "Sickle Cell Diseases")

**#3** - #1 AND #2

Refined by: LANGUAGES: ( ENGLISH )

Indexes=SCI-EXPANDED, SSCI, CPCI-S, CPCI-SSH, BKCI-S, BKCI-SSH, ESCI, CCR-EXPANDED, IC Timespan=2010-2020

**Search Strategy Documentation**

**Database: PubMed Provider: U.S. National Library of Medicine**

**Date(s) Searched: July 16, 2021 Date range searched: December 2020-July 2021**

**#1)** "food security"[tiab] OR "food insecurity"[tiab] OR food insecurity[mh] OR food security[majr] OR "food access"[tiab] OR "food hardship"[tiab] OR "food availability"[tiab] OR "food assistance"[tiab] OR "food desert"[tiab] OR "food deserts"[tiab] OR food deserts[mh] OR "food supply"[tiab] OR "food supplies"[tiab] OR "food insecure"[tiab] OR "food secure"[tiab] OR "food environment"[tiab] OR "food banks"[tiab] OR "food bank"[tiab] OR "food pantries"[tiab] OR "food pantry"[tiab] OR food supply[majr] OR "supplemental assistance nutrition program"[ti] OR "nutrition access"[tiab] OR supermarket[tiab] OR supermarkets[tiab] OR "grocery stores"[tiab] OR "grocery store"[tiab] OR "corner stores"[tiab] OR "convenience stores"[tiab] OR "food store"[tiab] OR groceries[tiab] OR "retail food environment"[tiab] OR “food retail”[tiab] OR "food marts"[tiab] OR "food mart"[tiab] OR “food outlets"[tiab] OR "food outlet"[tiab] OR “food environment”[tiab] OR “food environments”[tiab] OR “food mirages”[tiab] OR “fast-food”[tiab] OR “fast food”[tiab] OR food assistance[mh] OR "food aid"[tiab] OR "food stamp*"[tiab]

AND

**#2)** "sickle cell"[tiab] OR anemia, sickle cell[mh] OR "hemoglobin s disease*"[tiab] OR "Hbs disease"[tiab] OR "sickle cell anemias"[tiab] OR "sickle cell anemia"[tiab] OR "Sickle Cell Anemias"[tiab] OR "Hemoglobin S"[tiab] OR "Hemoglobin S Diseases"[tiab] OR "Sickle Cell Disorders"[tiab] OR "Sickle Cell Disorder"[tiab] OR "HbS Disease"[tiab] OR "Sickle Cell Disease"[tiab] OR "Sickle Cell Diseases"[tiab]

**#3)** #1 AND #2

Filters: Humans, English

**Database**: **Embase Provider: Elsevier**

**Date(s) Searched: July 16, 2021 Date range searched: December 2020 - July 2021**

**#1 -** "food security":ti,ab OR "food insecurity":ti,ab OR "food access":ti,ab OR "food hardship":ti,ab OR "food availability":ti,ab OR "food assistance":ti,ab OR "food desert":ti,ab OR "food deserts":ti,ab OR "food supply":ti,ab OR "food supplies":ti,ab OR "food insecure":ti,ab OR "food secure":ti,ab OR "food environment":ti,ab OR "food banks":ti,ab OR "food bank":ti,ab OR "food pantries":ti,ab OR "food pantry":ti,ab OR food supply:ti,ab OR "nutrition access":ti,ab OR supermarket:ti,ab OR supermarkets:ti,ab OR "grocery stores":ti,ab OR "grocery store":ti,ab OR "corner stores":ti,ab OR "convenience stores":ti,ab OR "food store":ti,ab OR groceries:ti,ab OR "retail food environment":ti,ab OR “food retail”:ti,ab OR "food marts":ti,ab OR "food mart":ti,ab OR “food outlets":ti,ab OR "food outlet":ti,ab OR “food environment”:ti,ab OR “food environments”:ti,ab OR “food mirages”:ti,ab OR “fast-food”:ti,ab OR “fast food”:ti,ab OR 'food insecurity'/exp OR 'food access'/exp OR 'food availability'/exp OR 'food security'/exp OR "food aid":ti,ab OR "food stamp*":ti,ab

**#2** - 'sickle cell':ti,ab OR 'sickle cell anemia'/exp OR "hemoglobin sc disease*":ti,ab OR "Hbs disease":ti,ab OR "Hemoglobin S Diseases":ti,ab

**#3** - #1 AND #2

**#4** - #4 AND [humans]/lim AND [english]/lim

3

**Database: CINAHL Provider: EBSCOhost Date(s) Searched: July 16, 2021 Date range searched: December 2020 – July 2021**

**#1** - (MH "Food Security") OR "food security" OR (MH "Food Assistance") OR "food security" OR "food insecurity" OR "food access" OR "food hardship" OR "food availability" OR (MH "food supply") OR "food assistance" OR "food desert" OR "food deserts" OR "food supply" OR "food supplies" OR "food insecure" OR "food secure" OR "food environment" OR "food banks" OR "food bank" OR "food pantries" OR "food pantry" OR "food supply" OR "supplemental assistance nutrition program" OR wic OR "nutrition access" OR supermarket OR supermarkets OR "grocery stores" OR "grocery store" OR "corner stores" OR "convenience stores" OR "food store" OR groceries OR "retail food environment" OR “food retail” OR "food marts" OR "food mart" OR “food outlets" OR "food outlet" OR “food environment” OR “food environments” OR “food mirages” OR “fast-food” OR “fast food”

**#2** - "sickle cell" OR (MH "anemia, sickle cell") OR "hemoglobin s disease*" OR "Hbs disease" OR "sickle cell anemias" OR "sickle cell anemia" OR "Hemoglobin S" OR "Hemoglobin S Diseases" OR "Sickle Cell Disorders" OR "Sickle Cell Disorder" OR "HbS Disease"

**#3** - #1 AND #2

Filters: English Language, Human

**Database: Cochrane Database of Systematic Reviews Provider: John Wiley & Sons**

**Date(s) Searched: July 16, 2021 Date range searched: December 2020 - July 2021**

**#1 -** "food security" OR "food insecurity" OR [mh "food insecurity"] OR "food access" OR "food hardship" OR "food availability" OR "food assistance" OR "food desert" OR "food deserts" OR "food supply" OR "food supplies" OR "food insecure" OR "food secure" OR "food environment" OR "food banks" OR "food bank" OR "food pantries" OR "food pantry" OR [mh "food supply"] OR "supplemental assistance nutrition program" OR "nutrition access" OR supermarket OR supermarkets OR "grocery stores" OR "grocery store" OR "corner stores" OR "convenience stores" OR "food store" OR groceries OR "retail food environment" OR "food retail" OR "food marts" OR "food mart" OR "food outlets" OR "food outlet" OR "food environment" OR "food environments" OR "food mirages" OR "food mirage" OR "fast-food" OR "fast food" OR "food aid" OR "food stamp*"

**#2 -** "sickle cell" OR [mh "anemia, sickle cell"] OR "hemoglobin s disease*" OR "Hbs disease" "Hemoglobin S" OR "Hemoglobin S Diseases"

**#3 -** #1 AND #2

Limits: Trials, English, Humans

**Database: Scopus Provider: Elsevier**

**Date(s) Searched: July 16, 2021 Date range searched: December 2020 – July 2021**

**#1 -** "food security" OR "food insecurity" OR "food access" OR "food hardship" OR "food availability" OR "food assistance" OR "food desert" OR "food deserts" OR "food supply" OR "food supplies" OR "food insecure" OR "food secure" OR "food environment" OR "food banks" OR "food bank" OR "food pantries" OR "food pantry" OR "supplemental assistance nutrition program" OR "nutrition access" OR supermarket OR supermarkets OR "grocery stores" OR "grocery store" OR "corner stores" OR "convenience stores" OR "food store" OR groceries OR "retail

4

food environment" OR "food retail" OR "food marts" OR "food mart" OR "food outlets" OR "food outlet" OR "food environment" OR "food environments" OR "food mirages" OR "food mirage" OR "fast-food" OR "fast food" OR "food aid" OR "food stamp*"

**#2 -** "sickle cell" OR "hemoglobin s disease*" OR "Hbs disease" "Hemoglobin S" OR "Hemoglobin S Diseases"

**#3 -** #1 AND #2

Limits: English, Humans

**Database: Web of Science Provider: Clarivate Analytics**

**Date(s) Searched: July 16, 2021 Date range searched: December 2020 – July 2021**

**#1** - TOPIC: ("food security" OR "food insecurity" OR "food access" OR "food hardship" OR "food availability" OR "food assistance" OR "food desert" OR "food deserts" OR "food supply" OR "food supplies" OR "food insecure" OR "food secure" OR "food environment" OR "food banks" OR "food bank" OR "food pantries" OR "food pantry" OR food supply OR "supplemental assistance nutrition program" OR "nutrition access" OR supermarket OR supermarkets OR "grocery stores" OR "grocery store" OR "corner stores" OR "convenience stores" OR "food store" OR groceries OR "retail food environment" OR “food retail” OR "food marts" OR "food mart" OR “food outlets" OR "food outlet" OR “food environment” OR “food environments” OR “food mirages” OR “fast-food” OR “fast food” OR "food aid" OR "food stamp*")

**#2** - TOPIC: ("sickle cell" OR "sickle cell anemia" OR "hemoglobin s disease*" OR "Hbs disease" OR "sickle cell anemias" OR "Hemoglobin S" OR "Hemoglobin S Diseases" OR "Sickle Cell Disorders" OR "Sickle Cell Disorder" OR "HbS Disease" OR "Sickle Cell Disease" OR "Sickle Cell Diseases")

("sickle cell" OR "sickle cell anemia" OR "hemoglobin s disease*" OR "Hbs disease" OR "sickle cell anemias" OR "Hemoglobin S" OR "Hemoglobin S Diseases" OR "Sickle Cell Disorders" OR "Sickle Cell Disorder" OR "HbS Disease" OR "Sickle Cell Disease" OR "Sickle Cell Diseases")

**#3** - #1 AND #2

Refined by: LANGUAGES: ( ENGLISH )

Indexes=SCI-EXPANDED, SSCI, CPCI-S, CPCI-SSH, BKCI-S, BKCI-SSH, ESCI, CCR-EXPANDED, IC Timespan=Dec 2020 – Jul 2021
